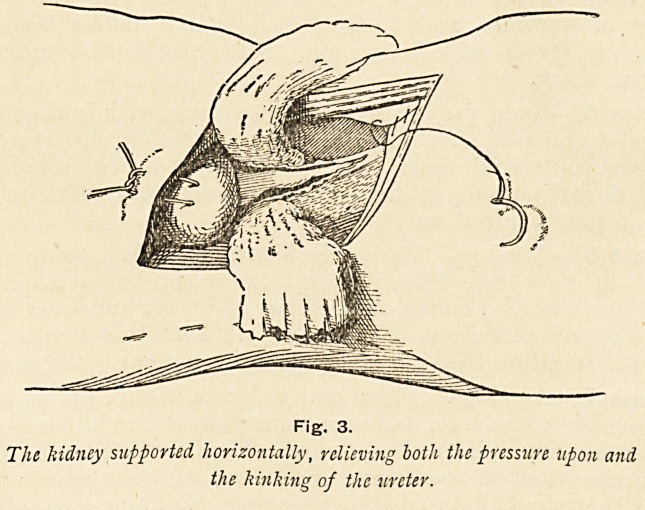# Nephropexy by Means of the Application of Strong Carbolic Acid and Temporary Support, with Report of Eight Cases

**Published:** 1905-03

**Authors:** T. Carwardine

**Affiliations:** Assistant-Surgeon to the Bristol Royal Infirmary


### NEPHROPEXY BY MEANS OF THE APPLICATION OF STRONG CARBOLIC ACID AND TEMPORARY SUPPORT,

### WITH REPORT OF EIGHT CASES.

BY

T. CARWARDINE, M.S., M.B. Lond., F.R.C.S.,

*Assistant-Surgeon to the Bristol Royal Infirmary.*

---

IN the *Lancet* for 1902[1] I briefly described a new method of nephropexy by means of the application of strong carbolic acid to the surface of the kidney, and as the method has been adopted by others, and has proved very satisfactory in my own hands, it may be well to give the details in this *Journal.*

My object in first attempting the method was to produce such a change in the surface of the kidney by chemical irritation that it would become covered with granulation tissue which would contract firm adhesions to the contiguous parts in a natural position, the adhesions so formed organising into firm fibrous tissue so as to ensure a secure anchorage for the organ. The results have borne out the expectations, and I have been able to watch the gradual development of the granulation tissue by actual inspection, its cohesion to the abdominal parietes, and, in two cases, I have been able to prove that the resulting fixation is so secure by the ultimate fibrous union that it becomes impossible to detach the kidney from the parietes with which it becomes firmly incorporated, except by cutting it away.

*The Method.*—I have employed the transverse or the oblique lumbar incision, the patient being on the side with the opposite loin supported by a pillow in order to increase the ilio-costal space; but the method could be carried out by the longitudinal dorsal incision if preferred. The muscles are in part divided and in part separated in the direction of their fibres. The last dorsal nerve may be identified with a little care, and held out of the way by a loop of catgut or silk and a pair of pressure-forceps. On complete division of the lumbar aponeurosis the

[1] *Lancet*, 1902, i. 1822.

outer fatty capsule appears and usually bulges into the wound when the kidney is pressed upon from the front. This is caught up by two pairs of forceps, placed far back as near the erector spinæ as possible, and divided between them. Any excess of this loose peri-renal fat should be excised. Occasionally it will be necessary to divide a few fibres of the erector spinæ, or of the quadratus lumborum when this latter is of unusual width. The finger is then passed into the wound to identify the kidney while the assistant presses on the abdomen in front. The kidney is then stripped of its surroundings as far as possible *in situ*. It is common in cases of wandering kidney, probably as the result of attacks of inflammation, to meet with strong

Fig. 1.

*Lower pole of kidney supported by a gauze sling.*

fibrous bands, some of which may need the scissors or scalpel for their division. Occasionally the whole hand must be inserted into the wound to free the upper pole of the kidney. Then the organ is brought carefully on to the loin by its lower pole, the body and upper pole following. The clearing of the kidney is then completed by finger and scissors, if necessary, until the renal pelvis is plainly visible. Gauze pads are then placed in the wound, and the whole surface of the exposed kidney is thoroughly painted with strong liquid carbolic acid two or three times by means of a swab held in pressure-forceps. The kidney becomes whitish in colour and sticky to the touch. The upper pole in particular should receive thorough treatment. The gauze is then removed from the wound and the kidney replaced into its normal position in the hypochondrium, first insinuating its upper or anterior pole. The centre of a strip of iodoform gauze, some 18 inches long, is next placed around the lower pole of the kidney, the ends remaining out of the wound. (Fig. 1.) Its object is simply to act as a loose temporary sling for the kidney during the granulating stage, and after the anterior part of the wound has been sutured a second piece is inserted lightly down to the kidney, and over this the two ends of the first piece are tied loosely together. This securely anchors the kidney for the time, and prevents any possibility of its dropping out of place. Plenty of absorbent antiseptic dressing is applied, a firm pad of absorbent wool placed over the hypochondrium in front, and the whole secured by a carefully-adjusted binder. I formerly employed through and through sutures of silkworm gut for the wound, but now prefer to suture in layers, employing continuous catgut sutures for the muscular and aponeurotic layers and silkworm gut for the skin. The reason for this more accurate suturing is that if through and through sutures be employed the resulting scar is apt to be painful during lateral and rotary movement of the body, when, in the natural condition of the parts, the various planes should slide upon one another. The restoration of this condition is favoured by suturing in layers.

*Pathology and After-treatment.*—During the first twenty-four hours there is a free discharge of watery serous fluid, having a slightly urinous odour, and at the end of this time the wound should be dressed and the surrounding skin cleaned. The two ends of the sling of gauze are untied, to be retied later after the blood-stained packing-gauze is replaced by a fresh piece. By throwing a light into the wound the kidney will be seen to be more opaque and white on the surface of the visible lower pole than at the time of operation, it moves slightly up and down with respiration, and when touched with the finger it feels firm and sticky, like damp wash-leather. The wound may be dressed every other day during the first week, the gauze packing being renewed when necessary. After a few days an excess of lymph will be observed on the surface of the kidney. At the end of a week the anterior part of the wound should be found healed by first intention and the stitches or continuous superficial suture may be removed. The surface of the kidney is by this time redder than at first. About the seventh day small reddish points or islands of granulations are visible on the renal surface, and by about the tenth day some of these may be seen to have coalesced with contiguous granulations on the abdominal parietes, forming glistening gelatinous strands uniting the two together, and the kidney shows less tendency than formerly to move with respiration. The sling of gauze may be left for ten days or a fortnight, or longer—in one case I left it for three weeks without ill effect. By this time it will have become loosened, and by cutting the lower end short and pulling on the upper end whilst coaxing the lower end round the pole of the kidney with the finger, it may be removed with but slight pain to the patient. I have several times shown that if the finger be placed upon the kidney at this stage it is impossible to dislocate it, firm union having already taken place, and the kidney being secured without any sutures to fix it. From this time onwards the surface of the kidney and the posterior angle of the wound granulate together in one. In the first two cases I used sutures for temporary support, in the remainder I have employed the temporary gauze sling for the purpose. In one instance (Case 6) a special suture was employed to sustain the kidney in a horizontal position and correct a valvular kink in the ureter which persisted otherwise. The method lends itself to this deliberate rotation of the kidney, a project which would be worth consideration in future when the question of ureteroplasty or neo-anastomosis arises.

The recovery in each of the eight cases was perfectly satisfactory, and in one case I had the opportunity of verifying the results after the lapse of twenty months. It was one of the earlier cases in which I had employed through and through sutures for the wound. After some months the scar became painful, and I determined to incise the scar and re-suture in layers. In doing so the scalpel entered the kidney substance for about half an inch in depth before I recognised it by the radial lines of tubules visible on the cut surface, so firmly and indistinguishably was the kidney united to the surrounding parts; indeed, it was with difficulty that the outer limit of the kidney could be separated from the fibrous tissue around. Moreover, the kidney substance visible was paler and firmer than usual, and scarcely bled, as though a certain amount of fibrosis had taken place by extension from the capsule along the trabeculæ. Another patient died two years subsequently from a perforated gastric ulcer, and has furnished similar evidence, which will be referred to subsequently.

*Advantages of the Method.*—In the first place it seems to be a certain method as compared with the earlier methods by simple suture in which there is a great tendency to relapse. So great was this tendency with the earlier suture methods that many surgeons formerly advised against the performance of nephropexy. From a careful examination of the cases treated by the method here described during the healing process, I can scarcely conceive that the kidney can again become loose. In the second place it is probable that there is a greater measure of safety by this method. The present mortality cannot be considered satisfactory for a condition which, in itself, is not fatal. Mr. Henry Morris, whom I have personally assisted at many kidney operations, estimates the average mortality at about 2 per cent.[1] This is nearly identical with the mortality of 14 in 846 cases collected by Edebohls.[2] But one must remember that the results of the average surgeon are apt to be less successful than those of surgeons with more operative experience and his results less likely to be published. In the suture methods, and when the capsule is freely stripped off, there may be some extravasation of urine into the wound, thus increasing the risk. In the former, also, there is a foreign body introduced into the kidney, for all now agree that if the kidney be sutured the sutures should pass well into its substance; whilst in the latter, though the sutures may be only passed through the detached capsule, this probably becomes dead tissue, and a favourable nidus for suppuration should infection occur. Further, by the method here described the kidney is restored to its normal

[1] H. Morris, *Surgical Diseases of Kidney and Ureter*, vol. i., 1901, p. 134.
[2] *Ann. Surg.*, 1902, xxxv. 179.

situation; this is somewhat difficult to obtain with the suture methods, which bring the body of the kidney below the costal margin, and Edebohls goes so far as to deliberately fix the kidney in an abnormal position midway between the costal margin and the iliac crest.

The following cases were all females with the kidney mobile on the right side:—

**Case 1.**—Aged 24. Had had paroxysmal pains as long as she could remember, with nausea, vomiting, and dyspepsia for two years. Reported after eighteen months to be "quite well with no symptoms."

**Case 2.**—Aged 18. Paroxysmal pains for two years, accompanied by nausea, constipation, and frequent micturition. She gained much flesh after operation, and left well and free from pain. Subsequently lost sight of.

**Case 3.**—Aged 25. Had pains as if someone gripped her loin, accompanied by vomiting, increasing for six months. Remained quite well for nine months; then scar became tender and painful. This was incised twenty months after, and resutured in layers, with complete relief. Kidney then found inseparably fixed.

**Case 4.**—Aged 24. Unable to work with pains in the loin. Belt tried twelve months. Reported herself two years after. Then in service, and feeling very much better than she had been for a very long time. Stays tight and making a distinct mark.

**Case 5.**—Aged 33. Pains and tenderness, with lump. Could only sleep on one side. A year after operation she wrote: "I thought you would be pleased to know that it is going on quite well. I feel no pain in side or scar unless I overwork or walk, then it passes after rest."

**Case 6.**—Aged 33. Very severe pain since first acute attack, due to lifting a bed. Intermittent tumour and polyuria. Kidney fixed horizontally, owing to kinking of ureter and overlapping by lower pole of kidney. (Figs. 2 and 3.) Seen two months afterwards; condition then satisfactory. Subsequently untraced.

**Case 7.**—Aged 47. Pains and sickness, which her physician was unable to relieve, and therefore placed her under my care for nephropexy. Left almost cured of her trouble, and glad she had undergone the operation. Reported eighteen months afterwards that she had lost her sickness and was on the whole very much better.

*Subsequent History.*—Two years after the operation the patient was admitted for perforated gastric ulcer, and during the operation for this the kidney was found to be securely fixed. The patient died, and the pathologist reported—that he had to cut the kidney out with a knife owing to its secure attachment; that the capsule of the kidney, which would not peel off, was of

Fig. 2.
*Showing kinked ureter pressed upon by lower pole of a loose kidney.*

Fig. 3.
*The kidney supported horizontally, relieving both the pressure upon and the kinking of the ureter.*

almost cartilaginous hardness, and that the interior of the kidney was quite healthy. A point of interest, moreover, was that the fixed right kidney was at a higher level than the left one.

**Case 8.**—Aged 30. Unable to work for last six months with pains and nausea, and she had no relief from a belt during that time. Reported six months afterwards as free from pain, with kidney firmly fixed.

Although in the majority of cases, including the 50 per cent. of neurotic types and those for whom some appliance suffices to afford relief—an operation is contra-indicated—there still remains a distinct class which may receive almost complete relief by operation, and a cure of the concomitant symptoms, now that we can promise them a reasonably safe and certain method of nephropexy. And this applies particularly to the class of young women whose livelihood depends upon a capacity for physical exertion in comfort, and whose trouble dates from the time of some definite strain.

*The Application of strong Carbolic Acid followed by Alcohol.*—In the cases above described, and as the method was a new one, it was desirable to watch for any possible symptoms of carbolic acid poisoning. Beyond a transient darkening of the urine there was no indication of any constitutional effects referable to the application. In a personal communication upon a different topic, the late Dr. A. M. Phelps, of New York, called my attention to the fact that Dr. Seneca D. Powell[1] had discovered that alcohol is an antidote to pure carbolic acid. If this be so, there could be no objection to its application to the kidney after the painting with carbolic acid, and this I propose to do on a future occasion. Knowing the value of carbolic acid in causing adhesions for the kidney, I recently employed it in a similar way for the liver and omentum in an advanced case of hepatic cirrhosis, followed immediately by alcohol on the assumption of its antidotal effect. When the carbolic acid was applied to the surface of the liver it became white and sticky, just as the kidney does; but on the subsequent application of the alcohol there was a striking change, the opaque whiteness at once disappearing and the liver capsule becoming translucent again so that the lobules were once more visible through it. At the same time the sticky feeling was lessened. Whether this physical effect—when carbolic acid

[1] *Am. J. Surg. and Gynec.*, 1902, xv. 127.

and then alcohol are applied to a viscus—corresponds with an antidotal effect, remains to be seen. But it points to the possibility of using the method within the peritoneal cavity for the artificial production of adhesions, as in Morrison's (Talma's) operation, and I hope to have further opportunity of testing its utility for the purpose.

## Figures and Tables

**Fig. 1. f1:**
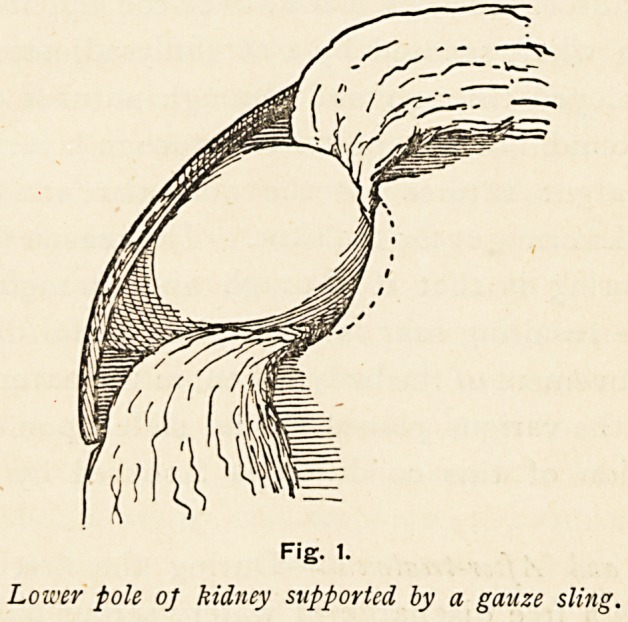


**Fig. 2. f2:**
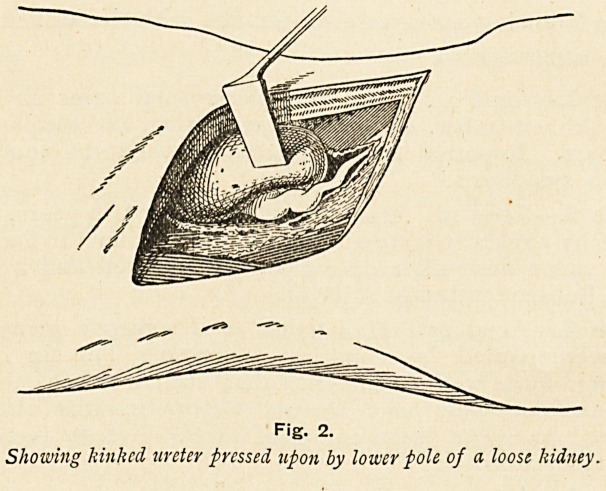


**Fig. 3. f3:**